# Quadratus Lumborum Block Reduced Postpartum Uterine Pain After Normal Spontaneous Delivery: A Prospective, Randomized, Double‐Blind, Controlled Trial

**DOI:** 10.1002/hsr2.72586

**Published:** 2026-05-31

**Authors:** Wen‐Shan Cheng, Yen‐Tin Chen, Wei‐Hsiang Chao, Chia‐Chih Liao

**Affiliations:** ^1^ Department of Anesthesiology Chang Gung Memorial Hospital Taoyuan Taiwan; ^2^ Department of Obstetrics and Gynaecology Chang Gung Memorial Hospital Taiwan; ^3^ College of Medicine Chang Gung University Taoyuan Taiwan

**Keywords:** level of interference, normal spontaneous delivery, quadratus lumborum block, ropivacaine

## Abstract

**Background and Aims:**

Normal spontaneous delivery is often accompanied by various types of pain; postpartum uterine pain can persist for 2 or 3 days after childbirth. The quadratus lumborum block (QLB) is a relatively safe, cost‐effective, and efficacious pain management strategy used in various abdominal surgeries. The present study aimed to determine the effectiveness of QLB in relieving postpartum uterine contraction pain in the immediate 48 h postpartum period.

**Methods:**

Sixty‐six women, who normally and spontaneous delivered between May 2022 and August 2022, were randomly assigned to undergo either QLB with 0.375% ropivacaine or QLB with pure normal saline for postpartum uterine pain relief. Primary outcome was the severity/degree of uterine pain according to the visual analog scale (VAS) score. Pain interference impacting participants' activities of daily living before and 1, 6, 12, 24, 36, and 48 h after intervention was a secondary outcome.

**Results:**

Those in the ropivacaine group required significantly fewer rescue analgesics and exhibited substantially lower mean VAS pain scores at rest and during activity, and a lower level of pain interference when compared with those in the normal saline group (*p* < 0.001) at 1, 6, 12, 24, 36, and 48 h after QLB.

**Conclusion:**

QLB was a valuable option for managing postpartum uterine pain after normal spontaneous delivery. It offered the benefits of stable hemodynamics, cost‐effectiveness, simplicity, and less pain interference with the activities of daily living, and minimal discomfort during the treatment process, with very few negative side effects.

**Trial Registration:** study registered with the clinical trials registry website (12/05/2022). ClinicalTrials.gov identifier: NCT05371015.

## 1 Introduction

Normal spontaneous delivery is often accompanied by various types of pain. Postpartum uterine pain can persist for 2 or 3 days after childbirth, which can cause discomfort and distress for new mothers. It is a natural mechanism that returns the uterus to its pre‐pregnant state to prevent postpartum hemorrhage (PPH) [1]. It has been established that postpartum uterine pain can be more pronounced in multiparous women, those who receive uterotonic drugs to prevent PPH, and those who breastfeed [2].

Previous studies have aimed to relieve postpartum uterine pain through pharmaceutical and non‐pharmaceutical approaches. Pharmaceutical therapies include acetaminophen, non‐steroidal anti‐inflammatory drugs (NSAIDs) [3], opioids [4], and herbal therapies. Non‐pharmaceutical therapies include massage, heat or cold packs, acupressure [5], and transcutaneous electrical nerve stimulation (TENS) [6, 7]. However, pharmaceutical therapies are often associated with limited effect on reducing visceral pain [8] or may cause other side effects [9, 10], while TENS can sometimes cause additional pain and discomfort during the treatment course [6]. With advances in ultrasound‐guided injection techniques, nerve blocks have become a major method for post‐operative pain control. The quadratus lumborum block (QLB) is a relatively safe, cost‐effective, and efficacious pain management method used in various abdominal surgeries, including gynecological laparoscopic procedures [11] and cesarean sections [12], to reduce somatic and visceral pain.

We administered local anesthetics into the fascia of the quadratus lumborum (QL) muscle via an applied anterior QLB, which can deliver local anesthetics into the paravertebral space, similar to the fascia iliaca plane block, and provide a clear endpoint that may also enhance visceral analgesia [13, 14]. Visceral pain was the primary source of discomfort that this study aimed to address.

However, no previous study has addressed the advantages of QLB in relieving postpartum uterine contraction pain. As such, the present investigation aimed to determine the effectiveness of QLB in relieving postpartum uterine contraction pain in the immediate 48 h postpartum period.

## 2 Materials and Methods

### 2.1 Ethics Approval and Consent to Participate

This double‐blind, prospective, randomized trial, which adhered to CONSORT guidelines, was conducted between May 2022 and August 2022. Ethical approval for this study was provided by the Institutional Review Board of Chang Gung Memorial Hospital, Taoyuan, Taiwan (approval number: 202102060A3) on February 15, 2022, and registered with the Clinical Trials Registry website (registration number: NCT05371015, 12/05/2022). All procedures involving human participants were performed in accordance with the 1964 Helsinki Declaration and its later amendments or comparable ethical standards. Participants gave informed consent to participate in the study before taking part.

### 2.2 Patients

Sixty‐six women, who normally and spontaneously delivered at the Department of Obstetrics and Gynecology, Chang Gung Memorial Hospital, were enrolled in this randomized controlled study, and written informed consent was obtained from all participants (conducted from May 2022 to August 2022).

Patients participating in this clinical trial were pregnant females (age ≧ 20 years) who normally and spontaneously delivered, and experienced moderate to severe postpartum pain due to uterine contractions (visual analogue scale [VAS] ≥ 4 points) immediately thereafter, and provided informed consent. Exclusion criteria included hemostasis abnormality, signs of infection at the provisional QLB injection site, known allergy to the intervention medication, known analgesic(s) misuse, episiotomy pain believed to be more disturbing than uterine contractions, unwilling to complete the study‐related questionnaire, and body mass index ≥ 35 kg/m^2^.

Sixty‐six eligible women were enrolled in this study and randomly assigned to one of two groups using computer‐generated random numbers: QLB with 0.375% ropivacaine; or QLB with normal saline.

SAS version 9.4 was used to generate a random allocation of participants. A non‐biased observer, who was not involved with the study, placed the allocations into sequentially numbered sleeves (from 1 to 66). Members of the study team remained blinded to participant allocation until the trial was officially unblinded.

### 2.3 Intervention

Labor analgesia was administered according to standard clinical practice and was discontinued immediately after delivery. All QLBs were performed after completion of labor analgesia, following transfer of the patient to the post‐delivery recovery room. An ultrasound unit (Sonimage HS2, Konica Minolta, Tokyo, Japan) equipped with a convex probe (C5‐2) was used. The techniques used for QLB were previously described by Blanco et al. [15] and slightly modified by Krohg et al. [16]. The QLB was performed with the patient in a supine position with a slight lateral tilt using a pillow placed under the ipsilateral flank. The curvilinear transducer was placed in the mid‐axillary line between the costal margin and the iliac crest. We used the “shamrock sign” approach, moving the probe posteriorly until the transverse process of L4, the psoas major (PM), the erector spinae (ES), and the quadratus lumborum (QL) muscles were identified. The needle was inserted from the anterior side in an in‐plane approach, passing through the QL muscle to reach the fascial plane between the QL and PM (the anterior QLB or transmuscular QLB) (Figure S1). After careful aspiration, a small volume of study medication was injected to confirm correct spread, followed by incremental administration of the remaining solution. The lean body weight (LBW) [17] of all participants was calculated initially. Participants in the ropivacaine group received 0.375% ropivacaine at a dose of 0.4 mL/kg (LBW) [18] per side, while those in the normal saline group received 0.4 mL/kg (LBW) of normal saline per side. Aspiration was repeated for every 5 mL of study solution injected for safety reasons.

All delivered women are required to be monitored in the specialized recovery area for at least 1 h following the third stage of labor. Crucially, this monitoring was conducted by delivery room nursing staff who were not part of the study personnel. During this period, patients were under the continuous supervision of specialized nursing staff and monitored using electrocardiogram, non‐invasive blood pressure, and SpO_2_ monitors. Patients were only transferred to the ordinary postpartum ward once they met the discharge criteria for hemodynamic stability.

### 2.4 Data Collection

The primary outcome was the severity/degree of uterine pain according to the VAS score [19]. The VAS was represented by a 10‐cm horizontal line, with two endpoints representing 0 (no pain) and 10 (pain as severe as it could possibly be). Participants evaluated current pain from uterine contractions before and 1, 6, 12, 24, 36, and 48 h after the intervention during rest and activity. The relating element of pain and sites of pain were also documented. VAS scores were recorded at rest and during activity at the study recruitment phase, and at times when participants required rescue analgesics after the intervention. Pain interference [20] impacting participants' activities of daily living before and 1, 6, 12, 24, 36, and 48 h after intervention was also documented as a secondary outcome. Pain interference level was determined according to a 0–10 rating in 7 daily domains (average activities, emotion, ambulatory function, day‐to‐day work, relationship with others, rest at night, and enjoyment of life); “0” represented least affected by pain and “10” is highly limited by pain. All adverse events were recorded.

### 2.5 Statistical Analysis

Baseline clinical characteristics of participants undergoing ropivacaine versus NS were compared using the independent sample *t*‐test for continuous variables or Fisher's exact test for categorical variables. VAS scores at rest and activity during the recruitment phase between the two treatment groups were also compared using the independent sample *t*‐test. Change in VAS scores, Brief Pain Inventory (each dimension and the mean pain interference level), and vital signs from recruitment phase (0 h) to later time points (1–48 h) between treatment groups were evaluated using generalized estimating equation (GEE) with normal distribution, identity link function, and exchangeable working correlation matrix. The GEE model included intercept, main effects of group and time points, and their interactions. In addition, VAS scores and Brief Pain Inventory were also compared at each time point between the treatment groups using the simple contrast from the same GEE model. All tests were two‐tailed, and differences with *p* < 0.05 were considered to be statistically significant. The primary objective of this study was to compare VAS scores between the two groups using repeated‐measures ANOVA (between‐subjects factor). The sample size was estimated based on the mean change in VAS scores from baseline reported by Dastjerdi et al. [21]. Based on these data, an effect size of 0.32 was calculated. Assuming a two‐sided α level of 0.05 and a statistical power of 80%, a total sample size of 46 participants was required. To account for potential attrition, the sample size was increased by incorporating an anticipated dropout rate of 40%, resulting in a final target enrollment of 66 participants (33 per group). Data analyses were performed using SPSS version 26 (IBM Corporation, Armonk, NY, USA).

## 3 Results

**Figure 1:**
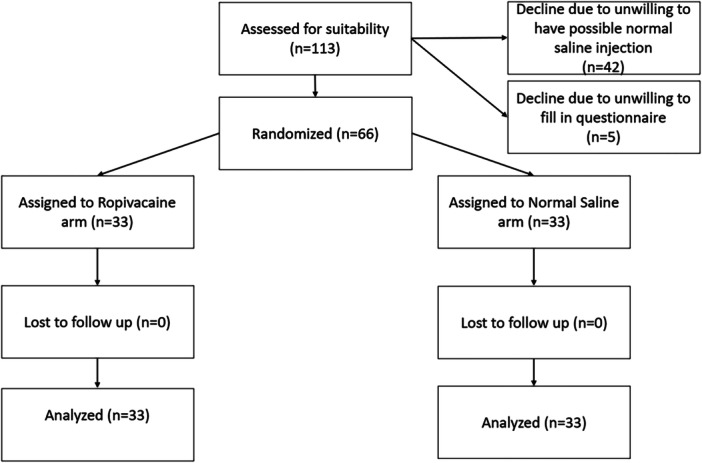
Flowchart of the delivered women enrolled in the study.

**Figure 2:**
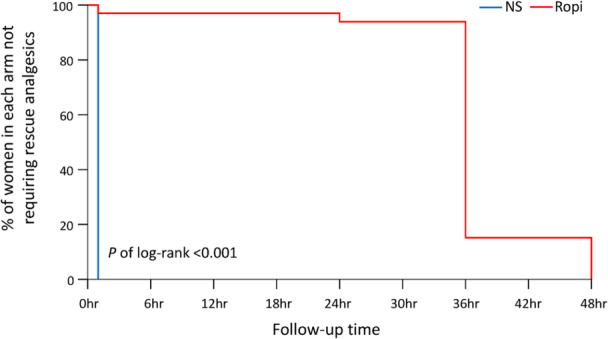
Proportion of women not requiring rescue analgesics in the ropivacaine and normal saline groups. The normal saline group showed a rapid decline within 1 h, whereas the ropivacaine group maintained analgesia‐free status for up to 24 h, with a decline after 36 h. Ropi as ropivacaine group. NS as normal saline group.

**Figure 3:**
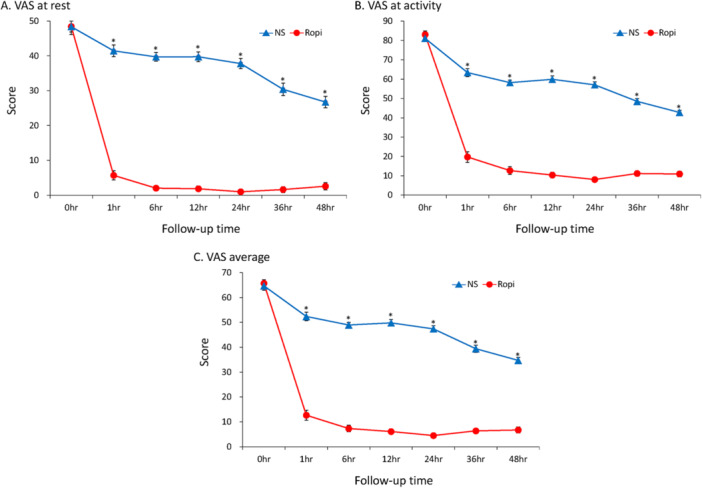
VAS scores at rest (A), activity (B), and average scores (C) at each time point in the ropivacaine and normal saline groups. *significant difference between the two groups. Ropi as ropivacaine group. NS as normal saline group. VAS, visual analogue scale. Data were presented as mean ± standard deviation.

**Figure 4:**
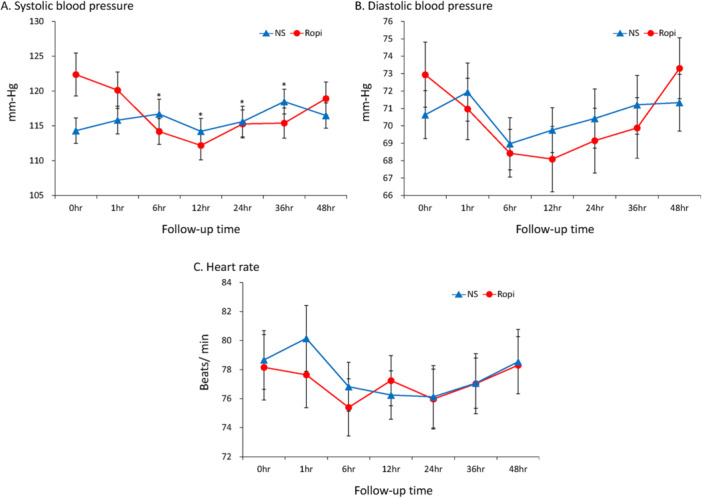
Systolic blood pressure (A), diastolic blood pressure (B), and heart rate (C) at each time point in the ropivacaine and normal saline groups. *significant difference between baseline time point (0 h) and follow‐up time points. Ropi as ropivacaine group. NS as normal saline group. DBP, diastolic blood pressure; HR, heart rate; SBP, systolic blood pressure. Data were presented as mean ± standard deviation.

Of 113 women who recently delivered and volunteered to participate in this study, only 66 were ultimately enrolled (Figure 1). There were 33 participants in each of the treatment groups. Delivery outcomes and characteristics were highly comparable between the two study groups (Table 1). Women in the ropivacaine group required significantly fewer rescue analgesics (Figure 2) and reported lower VAS scores than those in the NS group at multiple follow‐up time points, including 1, 6, 12, 24, 36, and 48 h after QLB (Table 2). Additional nonparametric analyses using the Mann–Whitney U test yielded results consistent with the primary analysis (Table S1). Similar patterns were observed for VAS scores at rest (Figure 3A), during activity (Figure 3B), and for mean VAS scores (Figure 3C). Additionally, the ropivacaine group experienced significantly lower painful sensation from uterine contractions after QLB compared with the NS group. Women in the ropivacaine group also experienced a significantly lower level of pain interference compared with those in the NS group across all eight categories (average activities, emotion, ambulatory function, day‐to‐day work, relationship with others, rest at night, enjoyment of life, and average level of interference) at multiple follow‐up time points, including 1, 6, 12, 24, 36, and 48 h after QLB (Table 3). The ropivacaine group exhibited significantly lower systolic blood pressure (SBP) between 6 and 36 h (i.e., 6, 12, 24, and 36 h) (Figure 4A) compared with its baseline (0 h). However, there were no statistical differences in hemodynamic stability of two study groups comparing with their own baseline time points (0 h) in terms of diastolic blood pressure (Figure 4B) and heart rate (Figure 4C). Importantly, there were no reported adverse events, except for mild discomfort from needle insertion, throughout the entire trial process.

**Table 1:** Characteristics data of the delivered women.

Variable	Ropi (*N* = 33)	NS (*N* = 33)	*p* value
Maternal age, weeks	32.1 ± 5.1	31.9 ± 4.4	0.856
Parity			0.588
0	12/33 (36.4%)	10/33 (30.3%)	
1	8/33 (24.2%)	12/33 (36.4%)	
≧2	13/33 (39.4%)	11/33 (33.3%)	
Height, cm	159.5 ± 4.7	161.9 ± 5.2	0.052
Body weight, kg	67.3 ± 6.9	70.7 ± 9.1	0.093
Body mass index, kg/m^2^	26.5 ± 2.8	27.0 ± 3.5	0.511
Pregnancy complication	6/33 (18.2%)	7/33 (21.2%)	0.757
Abortion	7/33 (21.2%)	9/33 (27.3%)	0.566
Tocolysis	13/33 (39.4%)	14/33 (42.4%)	0.802
Dysmenorrhea	18/33 (54.5%)	12/33 (36.4%)	0.138
Painless labor	29/33 (87.9%)	31/33 (93.9%)	0.392
Interval from delivery to enrollment, h	2.6 ± 0.8	2.9 ± 0.8	0.066
Established contractions to delivery, h	3.5 ± 2.1	4.3 ± 3.4	0.282
Duration of second stage of labor, min	49.2 ± 43.8	60.6 ± 62.5	0.393
Operative vaginal delivery	5/33 (15.2%)	7/33 (21.2%)	0.502
Postpartum hysterotonics			0.692
Piton	30/33 (90.9%)	28/33 (84.8%)	
Carbectocin	1/33 (3.0%)	1/33 (3.0%)	
Piton and carbectocin	2/33 (6.1%)	4/33 (12.1%)	
Blood loss, mL	217.3 ± 74.8	207.6 ± 25.4	0.483
Gestational age of newborn, weeks	38.4 ± 1.0	38.4 ± 0.7	0.885

*Note:* Ropi as ropivacaine group. NS as normal saline group.

Data were presented as frequency (percentage) or mean ± standard deviation.

**Table 2:** VAS scores during rest and activity at each time point.

Variable	Ropi (*n* = 33)	NS (*n* = 33)	Mean difference (95% CI)	*p* value
VAS rest				
0 h	48.4 ± 9.0	48.4 ± 12.8	0.06 (−5.37, 5.49)	0.982
1 h	5.7 ± 7.6	41.5 ± 9.7	−35.76 (−40.04, −31.47)	<0.001
6 h	2.0 ± 4.0	39.7 ± 7.2	−37.73 (−40.60, −34.86)	<0.001
12 h	1.8 ± 4.5	39.8 ± 8.0	−37.91 (−41.12, −34.70)	<0.001
24 h	1.0 ± 3.1	37.8 ± 8.5	−36.82 (−39.97, −33.67)	<0.001
36 h	1.6 ± 4.9	30.4 ± 10.1	−28.79 (−32.68, −24.90)	<0.001
48 h	2.6 ± 6.0	26.7 ± 9.6	−24.15 (−28.10, −20.20)	<0.001
VAS activity				
0 h	83.0 ± 10.8	81.1 ± 10.1	1.94 (−3.21, 7.08)	0.444
1 h	19.7 ± 16.0	63.3 ± 11.9	−43.64 (−50.57, −36.71)	<0.001
6 h	12.7 ± 11.5	58.2 ± 7.5	−45.48 (−50.25, −40.72)	<0.001
12 h	10.4 ± 8.5	59.9 ± 10.0	−49.55 (−54.11, −44.98)	<0.001
24 h	8.1 ± 5.6	57.0 ± 8.5	−48.97 (−52.51, −45.43)	<0.001
36 h	11.2 ± 7.6	48.5 ± 8.3	−37.27 (−41.18, −33.36)	<0.001
48 h	10.9 ± 8.7	42.7 ± 6.5	−31.79 (−35.55, −28.03)	<0.001
VAS average				
0 h	65.7 ± 8.3	64.7 ± 10.3	1.00 (−3.59, 5.59)	0.665
1 h	12.7 ± 11.4	52.4 ± 9.8	−39.70 (−44.93, −34.47)	<0.001
6 h	7.3 ± 7.4	49.0 ± 5.9	−41.61 (−44.89, −38.32)	<0.001
12 h	6.1 ± 5.9	49.8 ± 7.7	−43.73 (−47.10, −40.36)	<0.001
24 h	4.5 ± 3.9	47.4 ± 7.2	−42.89 (−45.74, −40.05)	<0.001
36 h	6.4 ± 5.7	39.4 ± 8.3	−33.03 (−36.51, −29.55)	<0.001
48 h	6.8 ± 6.9	34.7 ± 6.9	−27.97 (−31.36, −24.58)	<0.001

*Note:* Ropi as ropivacaine group. NS as normal saline group.

Data were presented as mean ± standard deviation.

Abbreviation: VAS, visual analogue scale.

**Table 3:** The brief pain inventory at each time point.

	Average activities			Emotion			Ambulatory function			Day‐to‐day work		
Time	Ropi	NS	*p* value	Ropi	NS	*p* value	Ropi	NS	*p* value	Ropi	NS	*p* value
0 h	6.8 ± 1.6	7.2 ± 1.3	0.300	5.4 ± 1.6	5.5 ± 2.1	0.735	6.2 ± 1.8	6.2 ± 1.8	0.944	5.9 ± 1.8	5.8 ± 2.1	0.898
1 h	2.3 ± 1.2	6.2 ± 1.6	<0.001	1.4 ± 0.9	5.2 ± 2.1	<0.001	1.5 ± 1.6	5.8 ± 1.8	<0.001	1.5 ± 1.9	5.0 ± 1.7	<0.001
6 h	1.5 ± 1.0	6.2 ± 1.5	<0.001	0.8 ± 0.8	5.1 ± 1.7	<0.001	0.8 ± 0.9	5.2 ± 1.8	<0.001	0.7 ± 0.9	5.2 ± 2.0	<0.001
12 h	1.1 ± 0.8	5.8 ± 1.2	<0.001	0.6 ± 0.7	4.8 ± 1.7	<0.001	0.6 ± 0.7	5.1 ± 1.9	<0.001	0.3 ± 0.6	4.6 ± 2.1	<0.001
24 h	0.8 ± 0.8	5.0 ± 1.2	<0.001	0.4 ± 0.5	4.4 ± 1.5	<0.001	0.6 ± 0.7	4.3 ± 1.5	<0.001	0.3 ± 0.5	4.1 ± 1.9	<0.001
36 h	0.9 ± 0.8	3.8 ± 1.1	<0.001	0.5 ± 0.8	2.9 ± 1.5	<0.001	0.4 ± 0.7	2.8 ± 1.8	<0.001	0.3 ± 0.7	2.5 ± 1.9	<0.001
48 h	0.7 ± 0.9	3.2 ± 1.4	<0.001	0.4 ± 0.7	2.5 ± 1.6	<0.001	0.4 ± 0.7	2.3 ± 2.0	<0.001	5.9 ± 1.8	5.8 ± 2.1	<0.001

	Relationship with others			Rest at night			Enjoyment of life			Average level of interference		
Time	Ropi	NS	*p* value	Ropi	NS	*p* value	Ropi	NS	*p* value	Ropi	NS	*p* value
0 h	6.0 ± 2.1	5.6 ± 2.2	0.423	6.6 ± 1.8	6.7 ± 1.9	0.841	6.1 ± 2.1	6.5 ± 2.1	0.468	6.2 ± 1.3	6.2 ± 1.5	0.830
1 h	1.3 ± 1.7	4.7 ± 2.0	<0.001	1.7 ± 1.2	5.7 ± 1.6	<0.001	1.2 ± 1.2	5.3 ± 1.8	<0.001	1.6 ± 1.1	5.4 ± 1.5	<0.001
6 h	0.7 ± 0.9	4.9 ± 1.7	<0.001	1.0 ± 1.0	5.8 ± 1.6	<0.001	0.7 ± 1.0	5.2 ± 1.7	<0.001	0.9 ± 0.7	5.4 ± 1.5	<0.001
12 h	0.3 ± 0.6	4.4 ± 1.9	<0.001	0.7 ± 0.7	5.4 ± 1.5	<0.001	0.5 ± 1.0	5.5 ± 1.9	<0.001	0.6 ± 0.6	5.1 ± 1.5	<0.001
24 h	0.5 ± 0.7	3.6 ± 1.9	<0.001	0.5 ± 0.6	4.8 ± 1.2	<0.001	0.3 ± 0.5	4.4 ± 1.8	<0.001	0.5 ± 0.5	4.3 ± 1.3	<0.001
36 h	0.3 ± 0.6	2.3 ± 1.7	<0.001	0.6 ± 0.7	3.5 ± 1.1	<0.001	0.3 ± 0.6	2.8 ± 1.7	<0.001	0.5 ± 0.6	3.0 ± 1.4	<0.001
48 h	0.3 ± 0.7	1.8 ± 1.6	<0.001	0.5 ± 0.7	2.8 ± 1.1	<0.001	0.3 ± 0.5	2.0 ± 1.5	<0.001	0.4 ± 0.5	2.4 ± 1.5	<0.001

*Note:* Ropi as ropivacaine group. NS as normal saline group.

The level of interference was presented as mean ± standard deviation.

## 4 Discussion

The results of the present study demonstrated that QLB can serve as a potent clinical alternative for women who experience postpartum uterine pain shortly after delivery. Most of the current oral pharmaceutical agents for controlling postpartum uterine pain, such as paracetamol/NSAID, have a limited effect on visceral pain [8], and postpartum uterine pain is of visceral origin. The paracetamol/NSAID had a limited effect on visceral pain due to its complex mechanisms associated with it. Visceral pain goes beyond the inflammatory pathways primarily involved with paracetamol/NSAIDs. Visceral pain is regulated by many other types of mechanisms [22], including autonomic nervous system activation and sensitization of peripheral afferent and dorsal horn neurons. While NSAIDs can reduce prostaglandin‐mediated inflammation, their analgesic effects on visceral pain are less significant compared to somatic pain. This limitation necessitates the use of other analgesic strategies for effective visceral pain management. The NS group had significantly higher VAS scores even though after having paracetamol/NSAID as pain relief during rest, activity and mean. (Figure 3A,B,C, respectively). The QLB also demonstrated the ability to reduce visceral pain in the literature [16, 23]. Furthermore, some non‐pharmaceutical methods may cause painful sensations during treatment [6] or require additional knowledge and training in complementary and alternative medicine, such as acupressure [5] and TENS [6, 7]. QLB offers an inexpensive, simple, and virtually painless treatment process for post‐partum uterine pain control, with very few negative side effects if administered with caution. In addition, no serious adverse events were reported. Most eligible participants who declined to join the study did so out of fear of being assigned to the NS group.

The VAS was chosen as the pain measurement tool because it is easily understood by participants and intuitive to use. While the VAS has been confirmed to be a reliable tool for pain assessment, it has its limitations, such as preset upper and lower caps, which should be considered when measuring ordinal and continuous data.

Although significant differences were found across all follow‐up time points (i.e., 1, 6, 12, 24, 36, and 48 h) in VAS scores at rest, during activity, and the mean VAS scores (Figure 3A,B,C, respectively), the absolute differences were most pronounced at 1, 6, 12, and 24 h. Kaplan–Meier analysis was also used to estimate the percentage of women in each group that did not require rescue analgesics. The NS group required rescue analgesics almost immediately after the intervention, while most of the ropivacaine group started to require rescue analgesics at the 36‐h follow‐up time point (log‐rank *p* < 0.001).

Hemodynamic stability is crucial for all pain control methods during the postpartum period due to the potential risk for PPH symptoms. Figure 4B,C reveal no significant differences in hemodynamic stability of the two study groups compared with their own baseline points (0 h). However, Figure 4A indicates that the ropivacaine group had significantly lower SBP at the 6, 12, 24, and 36 h follow‐up time points compared with baseline time points (0 h). Although the SBP values were close to the threshold for hypotension episodes (< 90 mmHg), it can be concluded that QLB for postpartum contraction pain exhibited a hypotensive tendency but did not trigger any hypotension episodes (< 90 mmHg) in this study.

While many studies have applied QLB as a method for pain control immediately after cesarean section [12, 24, 25, 26, 27], few have explicitly stated their choice of weight type for dosage. In this study, we selected LBW as the basis for dosing because LBW is significantly correlated with cardiac output [28] and is recommended as a safer tool for dosing estimation [29]. Pregnant women undergo various physiological and psychological changes [30], such as altered cardiac electrophysiology, increased cardiac output, reduced protein binding, and heightened sensitivity to local anesthetics, all of which contribute to an increased risk for local anesthetics systemic toxicity (LAST) [31]. Recent case reports have also described rare cases of QLB‐associated hematoma [32], particularly when the QLB injection site is close to lumbar arteries that run to the QL muscle posteriorly. Consequently, we adopted anterior QLB in this study to increase the distance from the potential hematoma and intravascular injection. Lateral QLB could potentially lead to hypotension more easily than other types of QLB due to bilateral sympathetic block resulting from paravertebral spread of local anesthetics. In summary, our approach aimed to minimize the risks for LAST, hypotension, and hematoma by using LBW instead of total body weight to account for physiological changes during pregnancy, using ultrasound guidance for peripheral nerve block (in this study: QLB), choosing ropivacaine over bupivacaine due to a higher cardiac/central nervous system ratio, and selecting anterior QLB instead of posterior and lateral QLB.

It is known that postpartum uterine pain progressively worsens as parity increases due to higher serum levels of oxytocin secretions [33, 34]. One limitation of this study was that there was only 1 grand multiparity (≧ 5 births) participant in the ropivacaine group, who did not require rescue analgesics until 36 h after undergoing ropivacaine, while there was no grand multiparity participant in the NS group. Due to the limited number of grand multiparity participants, the true effect of QLB in controlling postpartum uterine pain in grand multiparity remains to be determined. Unfortunately, due to ever‐decreasing fertility rates in Taiwan [35], the true answer to this question may long be left unanswered.

Pain affects patients' routine aspects of life, such as sleep, walking, emotions, and work. Thus, we implemented pain interference as a measure to evaluate the difference between the two groups. The ropivacaine group exhibited significantly lower levels of interference in all documented categories and at 1, 6, 12, 24, 36, and 48 h after the intervention. The true effect of proper analgesia in specific categories should be investigated further. Regarding emotion, we knew that patients with proper analgesia experienced lower levels of pain interference and fewer depressive symptoms than those with chronic pain [20]; however, does this also apply to postpartum women? Only further study will be able to address this question.

## 5 Conclusions

The results of the present study strongly support QLB as a valuable option for managing postpartum uterine pain in most common parities, offering the advantages of hemodynamic stability, cost‐effectiveness, simplicity, lower level of pain interference, and minimal discomfort during the treatment process. We are eager to explore further benefits of QLB in women seeking relief from postpartum uterine pain shortly after delivering.

## Author Contributions

**Wen‐Shan Cheng:** conceptualization, data curation, formal analysis, methodology, software, visualization, and writing – original draft. **Yen‐Tin Chen:** methodology, project administration, supervision, validation, writing – review and editing. **Wei‐Hsiang Chao:** methodology, project administration, supervision, validation, writing – review and editing. **Chia‐Chih Liao:** formal analysis, investigation, methodology, writing – review and editing.

## Funding

The authors have nothing to report.

## Consent

Informed consent was obtained from all subjects involved in the study.

## Conflicts of Interest

The authors declare no conflicts of interest.

## Transparency Statement

The corresponding author, Chia‐Chih Liao, affirms that this manuscript is an honest, accurate, and transparent account of the study being reported; that no important aspects of the study have been omitted; and that any discrepancies from the study as planned (and, if relevant, registered) have been explained.

## Supporting information


**Figure S1:** Ultrasound image of applying QL block.

**Table S1:** VAS scores during rest and activity at each time point.

## Data Availability

The data will be available upon reasonable request from the corresponding author.
