# Supplementary figures and images for: Quadratus Lumborum Block Reduced Postpartum Uterine Pain After Normal Spontaneous Delivery: A Prospective, Randomized, Double‐Blind, Controlled Trial
Source: Health Sci Rep. 2026 May 31;9(6):e72586. doi: 10.1002/hsr2.72586 (PMC13239790; doi:10.1002/hsr2.72586)

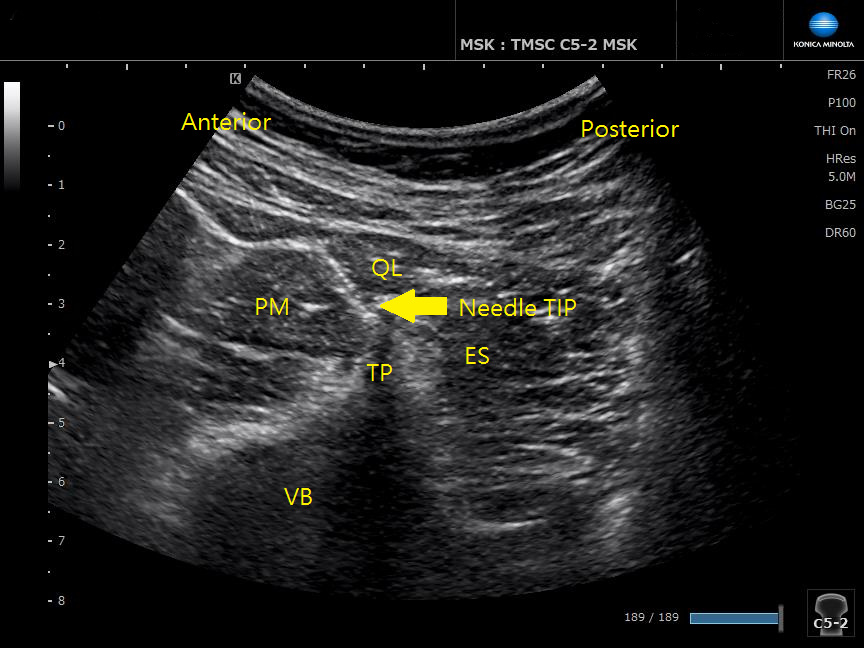

Supplement: Supplementary file 1 — Figure S1: Ultrasound image of applying QL block. [file HSR2-9-e72586-s002.tiff]
