# Supplementary material for: Quadratus Lumborum Block Reduced Postpartum Uterine Pain After Normal Spontaneous Delivery: A Prospective, Randomized, Double‐Blind, Controlled Trial
Source: Health Sci Rep. 2026 May 31;9(6):e72586. doi: 10.1002/hsr2.72586 (PMC13239790; doi:10.1002/hsr2.72586)
Supplement: Supplementary file 2 — Table S1: VAS scores during rest and activity at each time point. [file HSR2-9-e72586-s001.docx]

**Supplemental TABLE 1** VAS scores during rest and activity at each time point

| Variable | *Ropi*  (*n* = 33) | *NS*  (*n* = 33) | *P* value |
| --- | --- | --- | --- |
| VAS rest |  |  |  |
| 0 hr | 47.0 [43.0, 51.0] | 47.0 [40.0, 53.0] | 0.676 |
| 1 hr | 1.0 [0.0, 12.0] | 41.0 [35.0, 48.0] | <.001 |
| 6 hr | 0.0 [0.0, 1.0] | 41.0 [35.0, 43.0] | <.001 |
| 12 hr | 0.0 [0.0, 1.0] | 40.0 [34.0, 44.0] | <.001 |
| 24 hr | 0.0 [0.0, 1.0] | 36.0 [32.0, 43.0] | <.001 |
| 36 hr | 0.0 [0.0, 1.0] | 31.0 [23.0, 35.0] | <.001 |
| 48 hr | 0.0 [0.0, 2.0] | 25.0 [22.0, 35.0] | <.001 |
| VAS activity |  |  |  |
| 0 hr | 85.0 [73.0, 90.0] | 81.0 [73.0, 90.0] | 0.426 |
| 1 hr | 15.0 [7.0, 28.0] | 61.0 [55.0, 64.0] | <.001 |
| 6 hr | 10.0 [3.0, 18.0] | 57.0 [53.0, 63.0] | <.001 |
| 12 hr | 10.0 [3.0, 15.0] | 58.0 [54.0, 65.0] | <.001 |
| 24 hr | 8.0 [3.0, 11.0] | 57.0 [53.0, 63.0] | <.001 |
| 36 hr | 9.0 [6.0, 15.0] | 49.0 [44.0, 54.0] | <.001 |
| 48 hr | 9.0 [3.0, 17.0] | 42.0 [40.0, 48.0] | <.001 |
| VAS average |  |  |  |
| 0 hr | 67.0 [59.5, 71.0] | 65.0 [57.5, 71.0] | 0.476 |
| 1 hr | 8.0 [4.0, 22.0] | 51.0 [45.5, 57.5] | <.001 |
| 6 hr | 5.0 [1.5, 10.5] | 48.0 [45.0, 51.0] | <.001 |
| 12 hr | 5.0 [1.5, 8.0] | 48.0 [45.0, 54.5] | <.001 |
| 24 hr | 4.5 [2.0, 5.5] | 47.0 [43.0, 50.0] | <.001 |
| 36 hr | 5.5 [3.0, 7.5] | 38.0 [35.5, 46.0] | <.001 |
| 48 hr | 4.5 [2.0, 9.5] | 35.0 [31.0, 39.5] | <.001 |

*Note:* Ropi as ropivacaine group and NS as normal saline group;

VAS, visual analogue scale;

Data were presented as median [25th percentile, 75th percentiles].
